# Superior Colliculus Responses to Attended, Unattended, and Remembered Saccade Targets during Smooth Pursuit Eye Movements

**DOI:** 10.3389/fnsys.2016.00034

**Published:** 2016-04-12

**Authors:** Suryadeep Dash, Sina Alipour Nazari, Xiaogang Yan, Hongying Wang, J. Douglas Crawford

**Affiliations:** ^1^Center for Vision Research, York UniversityToronto, ON, Canada; ^2^Department of Physiology and Pharmacology, Robarts Research Institute, Western UniversityLondon, ON, Canada; ^3^Department of Psychology, Biology and Kinesiology and Health Sciences, York UniversityToronto, ON, Canada

**Keywords:** spatial updating, smooth pursuit, superior colliculi, attention, saccades

## Abstract

In realistic environments, keeping track of multiple visual targets during eye movements likely involves an interaction between vision, top-down spatial attention, memory, and self-motion information. Recently we found that the superior colliculus (SC) visual memory response is attention-sensitive and continuously updated relative to gaze direction. In that study, animals were trained to remember the location of a saccade target across an intervening smooth pursuit (SP) eye movement (Dash et al., 2015). Here, we modified this paradigm to directly compare the properties of visual and memory updating responses to attended and unattended targets. Our analysis shows that during SP, active SC visual vs. memory updating responses share similar gaze-centered spatio-temporal profiles (suggesting a common mechanism), but updating was weaker by ~25%, delayed by ~55 ms, and far more dependent on attention. Further, during SP the sum of passive visual responses (to distracter stimuli) and memory updating responses (to saccade targets) closely resembled the responses for active attentional tracking of visible saccade targets. These results suggest that SP updating signals provide a damped, delayed estimate of attended location that contributes to the gaze-centered tracking of both remembered and visible saccade targets.

## Introduction

Spatial updating is the ability to localize and act upon previously perceived targets when self-motion changes their location relative to body-fixed sensory organs (Klier and Angelaki, 2008; Sommer and Wurtz, 2008; Crawford et al., 2011; Medendorp, 2011). In the visual system, the double-step paradigm (i.e., a saccade to a visual target followed by another eye movement to a remembered target) is often used to demonstrate accurate visuospatial updating of goal-directed motor plans across various types of eye motion (Hallett and Lightstone, 1976; Herter and Guitton, 1998; Sommer and Wurtz, 2002; Baker et al., 2003; Daye et al., 2010; Dash et al., 2015). These experiments are typically done in complete darkness, but in real-world conditions both updating signals and visual signals are typically present. Very little is known about the functional contributions of updating signals to normal vision, or the way these signals differ/combine at the neurophysiological level. For example, Vaziri et al. (2006) showed that trans-saccadic updating improves manual pointing to visible targets, but this has not been studied in the oculomotor system and the neural correlates are unknown.

Visual receptive fields (RF) in the cortex and superior colliculus (SC) often become distorted just before and during saccades, but are then re-organized in their normal location relative to the final eye position: a process called predictive remapping (Duhamel et al., 1992; Walker et al., 1995; Umeno and Goldberg, 1997; Nakamura and Colby, 2002; Churan et al., 2012; Zirnsak et al., 2014; Mirpour and Bisley, 2015; Neupane et al., 2016). Viewed from the perspective of visual memory, remapping often results in the gaze-centered updating of visual activity (at a reduced level) during or just before saccade onset (Duhamel et al., 1992; Walker et al., 1995; Umeno and Goldberg, 2001; Inaba and Kawano, 2014), particularly when saccades place a remembered target within the RF of the neuron. In other experiments, it has been shown that visual responses may be augmented after a saccade (Ibbotson et al., 2008). However, a detailed comparison of updating and visual responses during saccades has not been done, perhaps because saccades are very brief and cause widespread suppression of visual responses (Burr et al., 1994; Phongphanphanee et al., 2011).

Spatial updating behavior is also observed across slow, continuous forms of eye motion such as full body translation and smooth pursuit (SP) eye movements of a moving visual target (Schlag et al., 1990; Ohtsuka, 1994; Zivotofsky et al., 1996; Baker et al., 2003; Medendorp et al., 2003; Blohm et al., 2005; Daye et al., 2010). In contrast to saccades, visual responses are not suppressed during SP. We, recently demonstrated that nearly all visually responsive neurons in the SC also show a transient memory response that is continuously updated the saccade goal during a preceding SP eye movement, i.e., the spatial memory response is shifted across the SC retinotopic map in opposition to the change in eye position (Dash et al., 2015). This response (henceforth called “updating”) was selectively enhanced when the memorized target was actively attended compared to the response when a distracter passively passed through the neuron’s RF. Thus, this provides an ideal experimental model for comparing the properties and functional interactions of visual and updating signals, but our previous study did not measure visual responses to sustained stimuli during SP.

In the present study we compared several important aspects of SC updating responses vs. visual responses during SP, including their relative magnitudes, spatio-temporal profiles, latencies, and dependence on attention. To do this, we recorded activity of visually responsive SC neurons during double-step SP-saccade task where animals were trained to make a saccade towards a remembered location after an intervening SP (SP updating task) and compared it with activity during exactly same task except that the saccade goal was visible throughout (SP visual task). In particular, we focused on comparing the dependence on attention, the spatio-temporal profiles, the magnitudes, and the temporal synchrony of these responses. We then tested whether the updating response might also contribute to vision when the stimulus is still present. In particular, we tested the hypothesis that the neural signals for attention-driven updating also contribute to the tracking of visible stimuli. We did this by comparing the responses during active memory updating, passive visual stimulation during pursuit, and the active attentional tracking of non-foveated visual targets during SP.

## Experimental Procedures

### Surgical Preparation and Electrophysiological Procedures

Two female rhesus monkeys, *Macaca mulatta*, (W and S) were prepared for head immobilization, two dimensional eye movements recordings and chronic electrophysiological recordings from SC. All surgical and experimental procedures were approved by the York University Animal Care Committee and were in compliance with the Canadian Council of Animal Care policy on the use of Laboratory animals. Monkeys underwent aseptic surgery under general anesthesia (isoflurane 1.5% and Ketamine 10 mg/kg). During surgery the animal underwent implantation of a stainless steel cylindrical tube (head post) and a plastic recording chamber (centered at 5 mm anterior and 0 mm lateral in stereotaxic coordinates, vertical approach with no angle) allowing easy access to SC. A circular craniotomy beneath the base of the chamber allowed access to both sides of the SC. In order to accurately measure (2D) eye position, the animal was implanted with one teflon coated braided stainless steel wire search coil (18 mm in diameter) sub-conjunctivally around the right eye. The head post, recording chamber and the socket attached to the search coil were affixed to the skull with the aid of dental acrylic and held in place with 13–15 stainless steel cortical screws. Animals were allowed 2 weeks of recovery following the surgery.

We, recorded extracellular neural activity from the SC with commercially available tungsten microelectrodes (FHC). A hydraulic microdrive (MO-90S, Narishige International USA) was used to lower single electrode into the SC based on stereotaxic coordinates. Detailed procedures for identification of the SC were described in our previous article (Dash et al., 2015), and recording sites have been confirmed histologically in both animals. Individual neurons were separated online based on template matching as provided by the Alpha–Omega Engineering Multi Spike Detector (MSD). Based on our criteria, all of our recordings were done within top 1.5 mm of the left SC in both animals. In general, during any session we encountered visual neurons first and then motor activity emerged as we went deeper, as expected from the known functional anatomy of the SC.

### Behavioral Procedures

Monkeys were trained to sit in a primate chair with their head immobilized and were fitted with a juice tube placed at their mouth for computer-controlled reward delivery (Crist Instruments). The monkeys were trained to generate the behavior of interest by rewarding them with units of fluid (juice or water), needed to satisfy their daily fluid requirements. Careful monitoring of fluid intake and body weight and supplementation of fluid outside the experiment if needed ensured that the animals were sufficiently hydrated at all the time. Monkeys were trained to keep their line of sight within an eye position window of 2–3° diameter centered on the fixation target (diameter 3 min of arc) presented on a computer monitor (Viewsonic P815, 20” screen diagonal, refresh rate 75 Hz, 1024 × 768 pixels) placed 40 cm in front of the monkeys in an otherwise completely dark room. We, used custom-designed software to present visual stimuli, control behavioral paradigms, and deliver juice rewards to the monkeys. As described in our previous article, we measured the rate of phosphor decay in our stimuli to confirm that this was complete approximately 1 s before the reported cell responses.

#### Memory Saccade Paradigm

During a typical trial the monkey fixated a small white dot (0.2°) in the center of the display (500–800 ms). While she fixated, a small (0.2°) white peripheral target was flashed for 200 ms. The monkey was required to maintain fixation throughout the target presentation period and also throughout the subsequent delay period (500–1000 ms; randomized variable delay between these two extremes). At the end of the delay interval the fixation target was extinguished and the monkey was required to make a saccade to the remembered location of the peripheral target. The monkey was rewarded if her eye position fixated within an 4° radius from the peripheral target location, within 500 ms of fixation target offset. Failure to perform this sequence of events resulted in the abortion of the trial (no reward) and the beginning of a new trial.

Using this paradigm, we screened the approximate location of visual and motor response fields (RF) for each recorded neuron in the right visual field, contralateral to our recording sites. Targets were presented in cardinal and oblique direction at 5°–20° eccentricities with 5° increments (20 different locations). We did not systematically map the complete RF of neurons as the purpose of using memory saccade paradigm was to get a quick, qualitative description of the neuron’s RF. Another block of memory saccade trials were carried out for a limited number of locations whose spatial coordinates were chosen based on the intended spatial configuration of the SP updating task. Memory saccade trials toward 7–9 different targets arranged in a linear array, which included the spatial boundary of the visual RF along the axis parallel to that of the subsequent SP ramp during SP updating task. Visual activity was sampled during a 100 ms interval that started 50 ms after visual target appearance (visual test) and it was compared to 100 ms period immediately preceding target presentation (control). The visual latency of neurons in our sample ranged between 50–70 ms. The motor activity was sampled in a 100 ms period starting 50 ms before saccade onset (motor test). To test if the neurons exhibited a memory response during the delay interval we defined a memory interval between 400–500 ms after visual target onset. This activity was compared to the same pre-stimulus control activity. All comparisons were made using Wilcoxon signed rank test and were deemed significant at *p* < 0.05. If only visual activity (for at least one stimulus location) was significantly different from its control, the neuron was deemed to be a visual neuron. Similarly, if only motor activity showed a significant difference, it was deemed to be a motor neuron. And if both visual and motor activity showed a significant difference, those neurons were deemed to be visuomotor neurons. The targets for which there was a significant visual response defined the entry point, exit point and center of visual RF (in one dimension) in the SP updating task. The approximate RF location of the neuron was used to ascertain the experimental configuration during the subsequent SP visual/updating task and was arranged in such a way that the saccade target (visual/memory target) corresponded approximately with the neuron’s RF somewhere along the SP ramp. The SP visual task and SP updating task were conducted in two separate blocks of trials whose order was randomized across the neural population.

#### SP Visual Task

**Figure 1:**
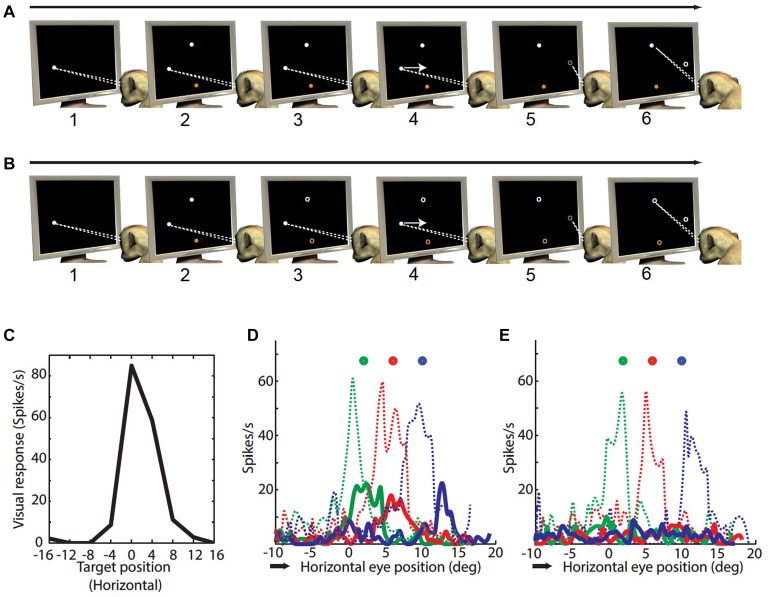
**Paradigms. (A)** Smooth pursuit (SP) visual task: (1) monkey fixated a white dot on the CRT monitor; (2–3) while fixating a peripheral target (white) and distracter (orange) appeared and continued to be visible; (4) the fixation dot started to move with a constant velocity (10°/s). Monkey followed the white dot with SP. The paradigm was configured in such a way that the target or distracter (white or orange dot) corresponded to the visual receptive fields (RF) of the neuron somewhere during the SP; (5) at an unpredictable time during SP the white SP target disappeared; (6) at this point the animal was required to make a saccade towards the visual target (white peripheral dot). **(B)** SP updating task: (1) monkey fixated a white dot on the CRT monitor; (2) while fixating a peripheral target (white) and distracter (orange) appeared for 200 ms and disappeared; (3) the animal kept the location of target in memory (memory target) and continued looking at fixation dot for another 300–500 ms; (4) the fixation dot started to move with a constant velocity (10°/s). Monkey followed the white dot with SP. The paradigm was configured in such a way that the memory target corresponded to the visual RF of the neuron somewhere during the SP; (5) at an unpredictable time during SP the white dot disappeared; (6) at this point the animal was required to make a saccade towards the memory target. The paradigm required the animals to continuously update its location during the SP. **(C)** One dimensional visual RF parallel to SP direction: the panel gives the average firing rate for visual stimulation (black) for various targets during memory saccade paradigm. The choice of these targets depended on the configuration of subsequent SP-visual/updating task. The targets corresponds to nine locations spanning from −16 to 16° in horizontal axis with a fixed vertical component of 5°. This visual neuron was most active at 0°/5° (horizontal/vertical), and was also active at 4°/5°. **(D)** Visual and updating responses to target: shows the neural activity in screen coordinates during SP visual/updating task (neural activity as a function of horizontal eye position). This is an example of updating across horizontal SP (from 10° leftward towards 18° rightwards; black arrow indicates SP direction) followed by a saccade to different visual/memory target (circles; targets were not visible during SP in SP updating task but visible during SP visual task). Colors indicate sets of trials associated with different visual/memory targets. **(E)** Visual and updating responses to distracter: same as **(D)** except visual and updating responses were collected when distracter corresponded with the RF of the neuron.

Monkey subjects were trained to pursue a moving visual target (white dot; 0.2°) and make a saccade towards a stationary visual target (white dot; 0.2°) while ignoring another stationary distracter stimulus (a 0.2° orange dot appearing in the mirror location across the SP ramp; Figure 1A). A typical trial started with the monkey aligning its line of sight to a target placed 10° right, up or down of straight ahead position. While fixating, the visual target and distracter appeared simultaneously while the animal kept fixating for another 300–500 ms. Following this the home fixation target stepped “backwards” before reversing direction and moving at a constant velocity (10°/s; this “step-ramp” paradigm was used to avoid catch up saccades during pursuit initiation: both animals had a pursuit initiation latency of approximately 150 ms so, we stepped the target back by 1.5°). Animals then followed the target with SP. At an unpredictable time during the SP the target disappeared. At this point the animal was required to make a saccadic eye movement towards the peripheral visual target while ignoring the visual distracter. Both visual target as well as the distracter was visible during the entire duration of the trial. Animals were only rewarded if the saccade landed within 5° (radius) of the visual target.

The direction of the SP ramp and the location of the visual targets/distracters were chosen based on the location of visual RF of the neuron. Each neuron was tested with a single ramp direction orthogonal to the vector derived from the peak of the response field obtained on-line in the laboratory. The basic experimental configuration for each SC neuron was arranged in such a way that the saccade target (visual target) passed approximately through the center of the neuron’s RF during the SP ramp. Furthermore, we ensured that the animal could not predict the end of SP ramp (and plan the subsequent saccade vector in advance) based on the location of the memory target. We did this by having three fixed SP ramp lengths (20°, 24° and 28°) and 3–5 different visual target/distracter locations relative to the end of each SP ramp length (= 9–15 different conditions per session). With this configuration most of the visual target locations (in screen coordinates) were associated with more than one SP ramp length, thereby decreasing the chance of predicting SP ramp end. Visual targets were linearly arranged along an axis parallel to the SP ramp and they were equally spaced from each other (4° separation). Animals were trained on this task until they were able to fulfill all of the above requirements and obtain a reward on >90% of the trials.

#### SP Updating Task

This task was essentially the same as our previous experiment (Dash et al., 2015) and was identical to the SP visual task, except that instead of a persistent visual target and distracter for the entire trial duration, the memory target and distracter were flashed for 200 ms at the beginning of the trial and the animals were required to keep the location of these target in memory and make a saccade to the remembered location after an intervening SP (for more details see Dash et al., 2015).

#### Data Analysis

Trial history, eye position records (sampled at 1.5625 kHz) and the timing of identified spikes were stored for later offline analysis. In addition, high-resolution (25 kHz) records of the electrode signal were kept for offline verification of the spike identification obtained online. The analysis was carried out using customized MATLAB programs (MATLAB, The MathsWorks Inc., MA, USA). The horizontal and vertical eye position records were smoothed using a Savitzky-Golay filter (window = 20 samples; polynomial degree = 4), which replaces the data points in the specified window by a polynomial fit of chosen order. We estimated the instantaneous firing rate of the recorded neurons with a continuous spike density function (SDF), generated by convoluting the spike train with a Gaussian function of *σ* = 10 ms width. We converted the discharge into SDFs in order to obtain the continuous description of neuronal activity.

As explained earlier, the experimental configuration for the *SP visual/updating task* was arranged for each SC neuron based on the approximate location of its visual RF. To quantitatively study the visual/updating response, data were aligned on the time point when the visual angle subtended by the instantaneous eye position and the visual target location corresponded to the entry point of the RF. The entry point of the RF was derived from the visual response during *memory saccade task*.

##### Single Unit Analysis

The average neural activity during the 500 ms period around the peak visual or updating response (inRF) was compared with a 500 ms period the immediately preceding the entry into the RF (outsideRF). If the activity in the inRF was significantly different from outsideRF, the neuron was deemed to show a visual response (Wilcoxon signed rank test; *p* < 0.05). The same time windows and statistics were used during the SP updating task to test if the neuron exhibited a significant updating response during SP updating task. We compared the visual/updating responses between the conditions when the target vs. the distracter was in the RF (Wilcoxon rank sum test; *p* < 0.05). For comparison of the magnitude of visual vs. updating responses for visual/memory target at single neuron level we compared the inRF activity for both groups (Wilcoxon rank sum test; *p* < 0.05).

In order to quantitatively analyze the correlation (*r*-value) between the visual response and updating response we cross-correlated the instantaneous visual response with the updating response at different time lags. The search range for lag estimation for best *r*-value ranged from −200 to 200 ms, i.e., visual and updating responses were moved relative to each other to get the optimal lag/lead where they are best correlated. The entire time period from 1 s before entry in to RF (outsideRF) to 1 s after RF entry (inRF) was used for the estimation of the best correlation coefficients and time lags.

##### Group Analysis

Group level comparisons (whether between visual vs. updating responses or target vs. distracter responses) were done using the averaged activity in inRF period for all the neurons (Wilcoxon signed rank test; *p* < 0.05).

## Results

We recorded 50 SC neurons in two rhesus monkeys (21 in Monkey S and 29 in Monkey W). Thirty nine out of 50 neurons were visually active and were considered for further analysis (Wilcoxon sign rank test; *p* < 0.05). Twenty one out of 39 visually active neurons also exhibited motor activity (visuomotor neurons), whereas 18 only showed visual responses (visual-only). Since the visual responses of visual-only and visuomotor neurons never showed any qualitative or quantitative differences in our subsequent analyses, we pooled their visual responses together for the analyses provided below and called these *visually active neurons*.

The bottom row of Figure 1 summarizes the continuous spatial updating responses for briefly presented memory targets that we reported previously and qualitatively compares these to the analogous visual responses to sustained stimuli. Figure 1C shows the task-relevant one-dimensional “slice” recorded of the RF of an example visual neuron. This figure shows the visual response for nine targets spanning horizontally from −16° to 16° (left to right separated by 4°) and with a constant vertical component of 5° relative to fovea (corresponding to the spatial range of visual or remembered targets that will pass through the neuron’s RF during our SP visual task or SP updating task, assuming for the moment that this RF is fixed relative to the eye). This neuron showed maximum response 5° vertically up relative to the fovea and also had significant response at 4° right/5° up relative to the fovea. Other locations did not evoke any significant visual response in this neuron.

Figures 1D,E plot neural activity for visual (dotted) and updating (solid) response as a function of horizontal eye position towards visual and memory targets and distracters, respectively. Figure 1D represents the visual and updating response towards three different targets spaced 4° apart from each other in the axis parallel to SP direction (circles with three different colors for three different set of trials presented randomly interleaved) during a 28° rightward SP starting from 10° left of center (arrow indicates the direction of SP). Figure 1E clearly shows the vigorous visual response (dotted traces) as soon as the location of the visual target started to overlap the RF of this neuron (4° right / 5° up). The peak visual response appears to be shifted slightly to the left (spatially) or before (temporally) the peak response that one would expect from the static RF. But the spatial resolution of our static RF maps was not high enough to analyze this quantitatively, and here we could not yet disambiguate spatial and temporal shifts. Therefore, we focused on comparison of visual vs. updating responses during SP.

**Figure 2:**
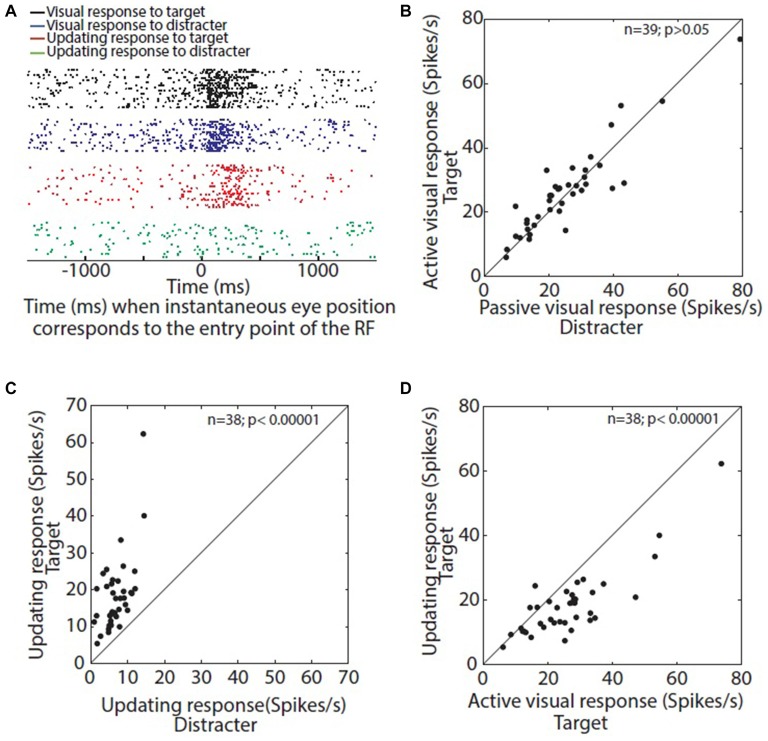
**(A)** Time aligned raster display of visual and updating response: black and blue raster’s corresponds to visual responses when target or distracter corresponded with entry point of the neuron’s RF, respectively. Red and green raster’s corresponds to updating responses for target and distracter, respectively. **(B)** Comparison of visual response for target and distracter****: average visual response for target is plotted as a function of visual response for distracter. **(C)** Comparison of updating responses for target and distracter: average updating response for target is plotted as a function of updating response for distracter. All the neurons show a higher updating response for target when compared with that for distracter. **(D)** Comparison of visual and updating responses for target: average updating response is plotted as a function of average visual response. Almost all the neurons show a higher visual response compared with updating response.

**Figure 3:**
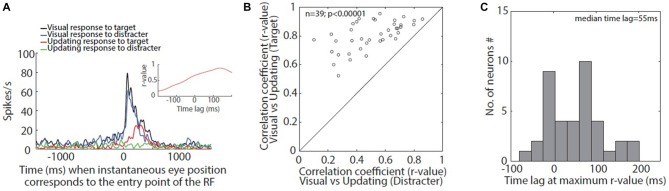
**(A)** Time aligned spike density function (SDF) of visual and updating responses: black and blue SDF corresponds to visual responses when target or distracter corresponded with entry point of the example neuron’s RF, respectively. Red and green SDF corresponds to updating responses for target and distracter, respectively. The inset shows the correlation between visual and updating response towards the target at different time lags. This neuron shows maximum correlation (*r*-value) when updating response lags behind the visual response by 137 ms. **(B)** Correlation between visual and updating responses: correlation between visual and updating response for target is plotted as a function of correlation for the distracter. All the neurons show a higher correlation between visual and updating response for target when compared with that for distracter. **(C)** Time lag for best correlation: shows the histogram representation of the time lag or lead when visual and updating responses showed maximum correlation across the sample of neurons. The median lag for the population was 55 ms, i.e., updating response lagged behind visual response by 55 ms.

**Figure 4:**
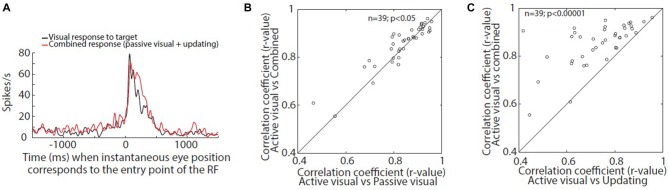
**(A)** Time aligned SDF of active visual response and combination of passive visual and active updating responses. **(B,C)** Correlation between active visual response and combined responses (passive visual response and updating response) compared to correlation between active and passive visual response **(B)** and correlation between active visual response and updating response **(C)** at zero time lag.

Consistent with our previous study, a clear updating response (solid traces) also emerged roughly when the location of remembered target coincided with the RF of the neuron (Dash et al., 2015). However, Figure 1D reveals that the updating response is much weaker than the visual response, and the updating response is slightly shifted in space (or time) compared to corresponding visual response. Figure 1E shows the visual and updating response towards the distracters. Whereas the visual response for distracter is equally vigorous as for a visual target, the updating response is absent. We will quantify these observations in more detail in the following sections (Figures 2, 3, 4).

### Strength of Visual and Updating Responses

Here we provide a more rigorous comparison of the magnitude of the four responses that we measured (visual vs. updating × active vs. passive). Figure 2A shows visual and updating responses in raster’s for the same visual neuron depicted in Figure 1 when instantaneous eye position corresponds to the entry point of the RF (4° right/ 5° up) during either SP visual task or SP updating task. This neuron shows a robust visual response towards both target (active visual response) as well as the distracter (passive visual response) when the eye brings the target into the neurons RF. At the group level, there was no difference between the visual response towards the target and the distracter (Figure 2B) but only 6/39 neurons showed a significantly different response in the active condition (Wilcoxon sign rank test; *p* > 0.05).

The example neuron in Figure 2A also exhibits a strong updating response towards target while no updating response towards the distracter. Consistent with our previous observation (Dash et al., 2015), almost all visually active neurons (38 out of 39) exhibited a significant updating response toward remembered location of target during SP updating task (Wilcoxon sign rank test; *p* < 0.05). Nineteen out of 39 visually active neurons exhibited significant updating response towards the distracter during SP updating task (Wilcoxon sign rank test; *p* < 0.05). At the single neuron level, 37 out of 38 updating neurons exhibited significantly higher updating response towards the target when compared with the distracter (Wilcoxon rank sum test; *p* < 0.05). The one remaining neuron did not show any significant difference but the average updating response for target was higher than that of the distracter. Figure 2C clearly shows that all the neurons are above the diagonal when mean updating response for target was plotted as a function of mean updating response for distracter. At the group level the updating response for target was significantly higher than that for distracter (Wilcoxon sign rank test; *p* < 0.000001). Thus, SC memory updating responses were much more sensitive to the influence of attention than SC visual signals during SP.

In the example neuron (Figure 2A), the updating response (red) is clearly weaker than the visual response (black). However, 20 out of 38 updating neurons showed a significantly higher visual response compared to updating response at single neuron level (Wilcoxon rank sum test; *p* < 0.05). Only one neuron showed a significantly higher updating response (Wilcoxon rank sum test; *p* < 0.05). However, overall the visual response was stronger across the population of 38 neurons (Wilcoxon sign rank test; *p* < 0.05; Figure 2D). The mean ratio of updating response and visual response across the population was 0.74, i.e., strength of updating response was approximately 74% of that of the visual response. Thus, at the overall population level, the active and passive visual responses were similar, both were significantly greater than the active updating response, and active updating (attended target) was significantly greater than passive updating (distracter).

### Correlation and Timing of Visual and Updating Responses

Here we provide a more rigorous analysis of the relative timing of the responses that we measured. Figure 3A shows the continuous spike density profile representation of visual and updating responses for the target as well as the distracter for an example neuron (same neuron as shown in Figures 1, 2) when the eye position corresponded with the entry point of the neuron’s RF (4° right / 5° up). It is clear in the figure that updating response towards target (red profile) lags behind the visual response (black profile), i.e., when compared to the visual response it starts to develop after a time lag. In order to quantitatively determine the time lag between the visual and the updating response and to explore how well the firing profile of visual response correlates with the updating response we cross-correlated both the profiles at a continuous range of time lags ranging between −200 ms to 200 ms. The visual and updating response in this example neuron showed a correlation coefficient (*r*-value) of 0.9 when the visual response was moved by 137 ms towards right. In other words, visual response led the updating response by 137 ms (or updating response lagged the visual response). The inset in Figure 3A shows the *r*-value for the full range of time lags analyzed.

Across the population of neurons, visual and updating response profiles towards target exhibited a strong correlation between each other (median *r*-value = 0.8433) which was always better than the correlation between visual and updating response profiles towards the distracter (median *r*-value = 0.2102; Wilcoxon sign rank test; *p* < 0.05; Figure 3B). Across population, the updating response lagged behind the visual response (median lag = 55 ms; Figure 3C).

### Active Visual Response as Combination of Passive Visual Response and Updating Response

In a natural environment, we often make movements towards target visible in peripheral vision after an intervening movement. Vaziri et al. (2006) have argued that the brain uses multiple sources of information for optimal response. In the SP active visual task (response to a continuous target), spatial attention, memory trace updating, and visual information were all available. If all of this information were used for tracking targets in the SC, this leads to the prediction that the SC response during this task should derive its features from both the updating response to an attended memory target and the passive sensory response to an unattended distractor. This can be observed in the example neuron shown in Figure 3A. The sharp initial response in the active visual task and the passive visual task were aligned, but the active response showed a more robust, longer lasting “tail” of activity. In contrast, the updating data lacked the sharp initial response, but had the longer lasting tail. Thus, in this neuron, the active visual response resembled the sum of the updating and passive responses.

To test this prediction quantitatively we combined the spike density profile of passive visual response and updating response and cross-correlated it with that of active visual response with zero time lag. If our hypothesis is correct then the combined response (passive visual response and updating response) should correlate better with active visual response at zero time lag compared with either of the individual responses. Figure 4A shows the continuous spike density profile representation of active visual (black) and combined responses (red) for an example neuron (same neuron as shown in Figures 1, 2, 3) when the eye position corresponded with the entry point of the neuron’s RF. It is clear that the tail end of the combined response coincide much better with active visual response when compared with passive visual response (Figure 3A). Upon cross-correlation of active visual response and combined response at zero lag we obtained a *r*-value (0.917) which was better than either cross-correlating active visual response with passive visual response (*r*-value = 0.900) or cross-correlating active visual response with updating response (*r*-value = 0.630). At the group level, correlation between active visual response and combined response was higher than either of the two contributing responses to combined response (Figures 4B,C; Wilcoxon signed rank test, *p* < 0.05). This supports our hypothesis that the active visual response to sustained stimuli is the sum of the passive visual response and the active updating response.

## Discussion

Many previous studies reported the existence of memory trace response in cortex and SC during inter-saccade interval in a double step saccade task or, when a saccade brings a previously seen visual stimuli into the RF (Duhamel et al., 1992; Walker et al., 1995; Umeno and Goldberg, 2001; Inaba and Kawano, 2014; Dash et al., 2015); but none of these directly compared spatial updating (or remapping) signals with the visual response for more than one spatial location. To our knowledge, this is the first study to systematically compare spatio-temporal response profiles of memory trace updating and corresponding visual response (and the influence of attention on these) in any brain area during an ongoing eye movement.

The current results confirm our previous SP updating study (Dash et al., 2015), in particular, the existence of gaze-centered updating signals in SC neurons with visual responses. But the current study also adds several new and important observations that required a direct comparison of updating vs. visual responses during SP. First, we found a high spatio-temporal correlation between updating and visual responses during SP. However, attention had a much stronger influence on memory updating than visual responses, the magnitude of updating response was weaker than the active visual response, and the majority of visually active SC neurons exhibited a lag in memory trace updating response when compared with the visual response. Finally, we obtained the highest spatio-temporal correlations when the sum of the passive visual response and the updating response was compared to the active visual response. We shall consider the significance of each of these results in more detail in the following sections.

### Similar Spatiotemporal Profiles in Updating and Visual Responses

The properties of visual RFs during saccades remain controversial (Zirnsak et al., 2014; Neupane et al., 2016), likely for the same reasons we outlined above (brief duration, widespread suppression). Here, we made a direct comparison of updating and visual response profiles when the animal was engaged in SP. Our paradigm allowed us to record visual and updating response through the entire span of neuron’s RF in one dimension; instead of just one spatial location typically used in previous studies (Duhamel et al., 1992; Walker et al., 1995; Umeno and Goldberg, 2001). In our previous comparison with static visual responses we found a high and significant correlation with updating responses in only 40% of visually responsive neurons. In the present study we find a high and significant correlation across the whole population of neurons. Thus, SC RF properties might be slightly modified by SP, but our data suggest that updating and visual responses share a very similar set of gaze-centered spatial inputs during SP. However, updating and visual responses also showed several differences, as follows.

### Differences Between Visual and Updating Responses During SP

#### Relative Influence of Attention

In our previous study, we observed that the SP spatial updating response is highly dependent on attention, i.e., to a saccade target vs. a distracter stimulus (Dash et al., 2015). However, we were not able to compare this to the influence of attention on visual signals in the same behavioral setting. Here, we were able to show that attention has a much bigger influence on updating responses than it does on visual responses in the SC. This is consistent with the notion that updating is an internally driven, cognitive process (Klier and Angelaki, 2008; Sommer and Wurtz, 2008; Medendorp, 2011). More generally, this is consistent with the notion that attention and working memory are tightly linked processes (Curtis, 2006; Prime et al., 2007; Luck and Vogel, 2013). However, to our knowledge across eye movements this has only been demonstrated at the neuronal level in a few recent studies, such as our experiments (Dash et al., 2015) and a more recent experiment on trans-saccadic remapping of memory trace (Yao et al., 2016).

#### Visual and Updating Response Magnitude

Post-saccadic memory trace responses are weaker than visual responses in the same neurons (Duhamel et al., 1992; Walker et al., 1995; Umeno and Goldberg, 2001), but this is very difficult to quantify during rapid eye movements because of the brevity of the sample period and widespread response suppression during saccades (Sommer and Wurtz, 2006; Zirnsak et al., 2014). These, however, are not limitations during SP updating. In our previous study, which utilized an indirect comparison between visual responses during static eye position and memory updating responses during SP, we estimated that a response magnitude ratio of 50%. Here, in a more direct comparison of responses during SP, we found a ratio of 75% between responses to active attended visual targets vs. updated memory targets. This is consistent with the need to internally reconstruct updating signals (Droulez and Berthoz, 1991; Keith et al., 2010), but it may also be somehow complementary to visual responses in normal lighting conditions. We will return to the latter topic below.

#### Temporal Lag in Spatial Updating Signals

Saccade-related remapping is well known to be anticipatory, to the extent that it begins even before the saccade (Duhamel et al., 1992; Walker et al., 1995; Umeno and Goldberg, 1997; Nakamura and Colby, 2002; Zirnsak et al., 2014; Neupane et al., 2016). Here, we used the cross-correlation between memory trace updating and visual response to reveal that the updating response was not predictive, but rather lagged the visual response by a median value of 55 ms (52 ± 63 ms; mean ± std). This suggests that during SP, SC updating signals represent a slightly delayed estimate of current target location relative to gaze.

This is consistent with previous behavioral studies in both humans and monkeys which showed an inability to account for subsequent smooth eye displacement when a saccade target was flashed near the end of pursuit (McKenzie and Lisberger, 1986; Gellman and Fletcher, 1992). Recently, Blohm et al. (2005) re-examined this question and concluded that a post-stimulus latency of >175 ms to generate an accurate saccade. Shorter latency saccades were directed towards the instantaneous retinal error subtended during target flash, as expected if updating were incomplete. Based on these results, Blohm and collegues inferred that a time lag was present in the extra-retinal pursuit signal for updating, as we observed here.

It is easy to explain why pursuit updating is not predictive compared to saccades: saccade planning signals are predictive and therefore can be used to predict updating, whereas SP is stimulus-velocity driven and unpredictable. However, it is harder to explain a lag. From the viewpoint of building an internal model of the world during pursuit one would ideally want a zero lag. Pursuit speed was constant across all the experimental sessions (= 10°/s) and the direction of the SP was constant in a given session and could provide predictive information on the SP trajectory. We only varied the length of the SP (in order to maintain unpredictability of the go signal and promote continuous updating of the saccade target). It may be that there is a hardwired lag in the system. Assuming that SP updating is driven by efference copies of an SP velocity signal (Blohm et al., 2006), these should not lag behind the actual SP. However, our updating responses were clearly attention-dependent, and such signals could involve significant lags (Egeth and Yantis, 1997). Again, another possibility is that these updating lags might somehow augment normal vision, as considered in the next section.

### Updating Signals During Normal Vision: Updating Spatial Attention?

Another possibility for the reduced gain and increased lag of updating responses relative to visual responses is that they serve some complementary function to vision in normal lighting conditions. For example, the presence of updating signals might augment and prolong visual signals, for improved attention and a better bridge it to action. It has previously been shown in the reach system that a combination of vision and updating provides more optimal behavior than either one alone (Vaziri et al., 2006), but to our knowledge, the current study is the first to show this occurring within actual neurons. In our final analysis, we tested such a prediction and found that active visual responses showed a high correlation with the sum of passive visual responses and updating responses. In our paradigm, this may have aided animals, even in the presence of a sustained visual target, to remain in a state of preparedness for a visuomotor transformation. In our paradigm, this only occurred at the “go signal”, at which time motor neurons began to show build up activity leading to a burst (see Dash et al., 2015, “Supplementary Figure 2”). Since the common element of our active visual and updating tasks was attention to a particular point in space, this suggests that pursuit updating signals are concerned with tracking (non-foveal) attended locations, whether these locations correspond to remembered stimuli or to stimuli that remain visible.

### Neural Mechanisms: Possible Sources of Attention, Memory and Updating Signals

**Figure 5:**
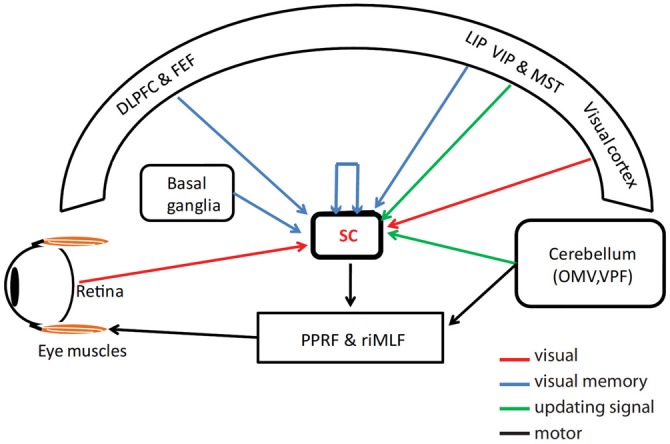
**Schematic representation of different sources of visual, memory and updating signals to superior colliculus (SC).** Visual signals (red arrows) is mostly contributed by visual cortex and retina but areas lateral intraparietal area (LIP), dorso-lateral prefrontal cortex (DLPFC) and frontal eye fields (FEF) also contributes visual signals to SC. The short term memory information (blue arrows) could reach SC from DLPFC, FEF, LIP or basal ganglia regions or maintained by intrinsic connections within SC. The eye velocity related updating signal (green arrows) could reach SC from various cortical areas (ventral intraparietal (VIP) and medial superior temporal (MST)) and/or cerebellar regions (oculomotor vermis and ventral paraflocculus). The black arrows indicate motor channels from SC to eye muscles through pendunculopontine reticular formation (PPRF) and rostral interstitial nucleus of medial longitudinal fasciculus (riMLF).

Humans and monkeys are able to accurately memorize a location in space and update the location after an intervening SP with the head mobile or immobilized (Schlag et al., 1990; Ohtsuka, 1994; Herter and Guitton, 1998; Baker et al., 2003; Blohm et al., 2005; Daye et al., 2010; Dash et al., 2015). Dash et al. (2015) identified a neural correlate of this memory trace response; continuous updating of a “hill” of activity in visually responsive neurons, corresponding to the gaze-centered location of a memorized target in the SC topographic map. Any brain area exhibiting above response should possess or receive several signals: first, visual inputs, to initiate the updating system and/or combine later with updating signals for active vision, second, attention-dependent mechanisms to hold the visual location in short-term memory (Goldman-Rakic, 1995; Baddeley, 2003, 2012), and third, access to an appropriate updating signal (Figure 5).

The visual inputs to the SC are well described and include both retino-tectal projections as well as projections from occipital and parietal cortex (May, 2006). Once such stimuli have activated the SC, many computational models have proposed that attended stimuli might be maintained by recurrent connections, both intrinsic to structures like the SC and between different regions, both during fixation and spatial updating task (Xing and Andersen, 2000; Keith et al., 2010). These signals could involve both intrinsic SC circuits as well as projections from cortical and sub-cortical regions including caudate nucleus, substantia nigra, dorso-lateral prefrontal cortex (DLPFC), frontal eye fields (FEF) as well as lateral intraparietal area (LIP; Hikosaka and Wurtz, 1983; Hikosaka and Sakamoto, 1986; Gnadt and Andersen, 1988; Funahashi et al., 1989; Goldman-Rakic, 1995; Constantinidis and Steinmetz, 1996; Compte et al., 2003; Armstrong et al., 2009).

What feedback information could be used as updating signal during SP updating? Based on behavioral observations, Blohm et al. (2006) proposed a model that used the delayed integration of eye velocity signal to obtain eye displacement signal prior to updating target location. Furthermore, consistent with our results, other computational modeling studies showed that when eye velocity was used as the updating signal, there was a continuous moving hill of activity across two dimensional topographic representation of visual space (Droulez and Berthoz, 1991; Keith et al., 2010). Various cortical and cerebellar regions, ventral intraparietal area (VIP), FEF, medial superior temporal area (MST), oculomotor vermis and ventral paraflocculus carry velocity signals which could be the source of updating signal and reach SC directly or indirectly (Fukushima et al., 1999; Ilg and Thier, 2003; Schlack et al., 2003; Medina and Lisberger, 2007; Dash et al., 2012). Finally, projections from visually responsive updating neurons in the SC to saccade motor neurons—both within the SC and other structures like FEF and LIP—would be required to initiate saccade activity in premotor burst neurons (Büttner-Ennever and Büttner, 1988; Crawford and Vilis, 1992; Moschovakis et al., 1996; Sparks, 2002).Together, our current results suggest that these circuits provide two interacting systems for optimal target tracking during pursuit: a completely internal attention-driven feedback loop for updating target memory (Dash et al., 2015) embedded within a vision-driven closed-loop feedback system.

## Author Contributions

SD and JDC designed the experiment, SD and SAN conducted the experiments and analyzed the data, XY and HW provided technical and surgical help. SD and JDC wrote the manuscript.

## Funding

This project was funded by the Canadian Institutes for Health Research. JDC was supported by a Canada Research Chair, and SD was supported by an Ontario Ministry of Research and Innovation Fellowship.

## Conflict of Interest Statement

The authors declare that the research was conducted in the absence of any commercial or financial relationships that could be construed as a potential conflict of interest.
